# The Scientific Temperance Federation

**Published:** 1908-01

**Authors:** 


### The Scientific Temperance Federation.

The great improvement in health and length of life that has followed the acquirement of better knowledge of hygiene and sanitation shows how powerful a means knowledge is for the correction of unhygienic living.

In the various efforts being made to extend knowledge of the essentials of hygienic living, there has come to be a recognized need of a central agency for the collection and dissemination of scientific findings concerning alcoholic drinks and other narcotics in their relations to individual and social welfare.

Many valuable books, pamphlets, papers and other studies in these fields have now an all too limited usefulness, merely because they are not kept on file, and their facts are not brought to the attention of the general public by some central independent agency.

There is need, therefore, of the assistance of a trained acquaintance with the publications and help of the disassociated workers which can at once refer the inquirer to the particular facts he wishes, and can turn all useful information on these subjects into every possible channel through which it can reach the people.

The Scientific Temperance Federation was organized December 21, 1906, to meet this need.

Its aim is to bring together the facts developed by scientific research and experience, making them accessible to all persons interested in the great questions of sobriety and hygienic living; to disseminate such facts in every possible way; to promote the hygienic and temperance instruction of the children and youth in the public schools.

It will enable the specialist to put his conclusions on file where they will be sought for and examined, and the student or individual worker to obtain desired information.

It will not supersede existing organizations, but will endeavor rather to unify and supplement them.

A wide field of usefulness is open to the Federation, while it presents varied opportunities for mutual assistance and co-operation.

The Federation presents a medium for widely disseminating hygienic truths. It offers access also to up-to-date collections of purely scientific research concerning the physiological, chemical and psychological relations of alcoholic drinks and other narcotics. To this end, it welcomes books, pamphlets, papers, etc, containing information on these topics.

For literature address Miss C. F. Stoddard, Corresponding Secretary and Treasurer, 23 Trull Street, Boston, Mass.

